# Remarks on the Diagnosis of Dislocations of the Shoulder Joint

**Published:** 1856-04

**Authors:** L. A. Dugas


*Remarks on the Diagnosis of Dislocations of the Shoulder Joint.*
By L. A. Dugas, M. D., &c.

The very frequent instances of permanent disability and deformity consequent upon the non-detection of dislocations of the Humerus, abundantly demonstrate the difficulty of their diagnosis, and would seem to demand at the hands of the profession a more careful study of the subject. The object of this paper is therefore to refresh the memory of those who will be more likely to read a periodical than to go back to their systematic treatises, and at the same time to suggest a means of diagnosis not referred to in works on Surgery, so far as my recollection serves me.

I will not stop to notice the various classifications proposed by authors, especially the French and Germans, who have perhaps multiplied varieties unnecessarily; but at once adopt that commonly admitted in this country. The head of the humerus may then be dislocated downward, into the axilla; forward, below the clavicle; and backward, upon the scapula. By far the most common of these accidents, is that in which the head of the bone is found in the axilla; whilst that in which the head of the humerus rests upon the scapula is so exceedingly rare, that many experienced surgeons have never seen a case of the kind. The reason of this relative difference is easily ascertained by reference to the anatomical arrangement of the parts; for although the so-called glenoid *cavity* is in reality almost a *plane* surface, which could of itself offer no impediment to dislocations in any direction, the head of the humerus is effectually prevented from escaping in certain directions by the strong arch which overhangs the articular surface of the scapula. This arch is formed by the acromion and coracoid processes and the intervening ligaments, fortified by the capsular ligament and the deltoid muscle. Now, while the anterior margin of this arch, formed by the coracoid process, does not descend below the upper fourth of the glenoid cavity, its posterior margin, consisting of the root of the acromion, comes down sufficiently low to protect almost, if not quite, one-half of the articular surface. The only space left, therefore, by which the humerus could escape backward, is that which intervenes between the acromion and the attachment of the great and strong tendon of the triceps extensor cubiti, which space is very small when compared with that between this tendon and the coracoid process. Hence the rarity of one accident and the frequency of the other.

But, to return to our immediate subject, the first consideration should be, not the signs or symptoms by which we may discriminate between the different dislocations of the shoulder, but rather the means by which we may determine whether there is any dislocation at all, or not. It is self-evident that if it can be ascertained that the head of the humerus is not in the glenoid cavity, it is displaced, or, in another word, *dislocated.* This fact can *usually* be determined by the depression observable below the end of the acromion process and beneath the deltoid muscle, both by the eye and by the hand pressed upon the shoulder. But there are many cases in which the fleshiness of the patient, or the tumefaction consequent upon the injury, are such as to render this element of diagnosis of little value, especially when we recollect that more or less depression would attend a fracture of the humerus near its head, and even a fracture of the neck of the scapula. Such a degree of depression as we might, by careful examination, detect in a fleshy person, or when there is much swelling, would therefore scarcely be deemed sufficiently positive in itself for practical purposes. It would become necessary to determine whether or not this depended upon the existence of a fracture. This might be done by endeavoring to produce crepitus, and by looking for the head of the bone in the localities in which it might be carried by dislocation. The sensation of crepitis and the absence of the head of the humerus from the axilla, from below the clavicle and from the dorsum of the scapula, would establish conclusively that the cause of the depression observed, is to be found in a fracture; whereas the detection of the head of the humerus in any other place than in the glenoid cavity would establish the existence of a dislocation as the cause of the depression in question.

Again: the existence of dislocation may be sometimes determined by the detection of the head of the os humeri in an abnormal situation; and this would be conclusive without reference to any depression below the acromion. But there are individuals whose embonpoint, or whose muscular development is such as to render this detection extremely difficult. Moreover, a fracture near the head of the humerus might be attended with such a displacement of the upper end of the lower fragment as to allow this to be taken for the head of the bone in the axilla, especially in individuals presenting the peculiarities just mentioned, or in cases attended with much swelling. It should be remembered, that the head of the humerus becomes more prominent and may be felt in the axilla when the elbow is elevated, although it may have escaped detection while the arm was allowed to hang down.

It is needless to dwell upon the value of the information to be derived from the history of the accident, of the degree or kind of disability experienced by the patient, of the change in the length of the arm and direction of the shaft of the bone, of the pain induced by carrying the elbow in different directions, &c. All these circumstances should of course be studied by the surgeon in doubtful cases; but neither of them can lead to anything positive.

From these reflexions, the necessary inference must be drawn, that a concurrence of circumstances can alone be relied upon in certain cases to make the diagnosis of dislocation clear and positive. The discovery, then, of some one, unequivocal, pathognomonic fact, or sign, by which a dislocation of this joint could in all cases be readily ascertained, must be a great desideratum. I believe that this will be found in the following maxim: if the fingers of the injured limb can be placed (by the patient or the surgeon) upon the sound shoulder, while the elbow touches the thorax, there can be no dislocation; and if this *cannot* be done, there *must* be a dislocation. I am aware that most writers indicate the difficulty of bringing the elbow in contact with the thorax as a symptom more or less marked in the several dislocations of the humerus, but it is obvious that they do not assign to it the degree of importance to which it is entitled when coupled with the injunction to place the fingers upon the opposite shoulder, as just enunciated. Indeed, most authorities merely mention the fact that the elbow cannot be approximated to the trunk *without pain*. I insist that the elbow cannot be brought against the thorax when carried sufficiently in front to allow the fingers to rest upon the sound shoulder, either by the patient or by the surgeon, not because this would be painful, but because it is physically impossible without a reduction of the dislocation or such a disruption of the shoulder, as no one would think of attempting. This becomes at once manifest by looking to the skeleton. With this before us, let us place the head of the humerus in the axillary region, and we observe that the elbow is at once thrown out from the trunk; that if we attempt to bring it towards the sternum, the upper portion of the shaft of the humerus will soon encounter the ribs, and that the rotundity of the thorax will effectually prevent any contact between the elbow and sternum, unless, by using the humerus as a lever, we rend asunder all the obstacle in the way of the rising of the head of this bone. Our maxim then holds good with regard to dislocations into the axilla. Let us now place the head of the humerus where it would be found in a forward dislocation; that is to say, below the clavicle. We will then perceive that the elbow is still removed from the trunk, and that it is moreover directed backward, thus presenting, perhaps even to a greater degree, all the difficulties just specified, if it be endeavored to carry the elbow against the sternum and the fingers upon the other shoulder. With regard to the dislocation backward, the impossibility of bringing the elbow and fingers in the positions indicated, becomes so manifest that it requires no comment.

Now, can any other accident than a dislocation offer these obstacles to placing the elbow and fingers in the above-mentioned position? I unhesitatingly answer, there is none. It is true, that a severe contusion may render this or any other motion more or less painful to the patient, and that he will aver that he cannot do as directed. But the surgeon will find no difficulty in accomplishing his design, especially if he proceed gently, so as not to encounter any muscular resistance, voluntary or otherwise. The same remarks apply with equal force to fractures of the upper end of the humerus or of the neck of the scapula. In all these cases the administration of an anæsthetic will save the patient some pain and facilitate the examination.

If, then, a dislocation of the humerus in any direction, whatever, renders it impossible to place the fingers and elbow simultaneously in the position stated, and that no other accident can produce this inability, the corollary is clearly that such an impossibility is pathognomonic of dislocation of the humerus; and, conversely, that the absence of this inability as clearly demonstrates the absence of dislocation.

Having in this manner settled the all-important question of the existence or non-existence of dislocation, it will not be found very difficult to determine the new position assumed by the dislocated head of the humerus. Indeed, the mere direction of the axis of the humerus will suffice in most instances to lead us directly to its head. This axis can be usually traced without difficulty, with the fingers, from the elbow up to at least six or eight inches; a length sufficient to allow the imagination to prolong it to the upper end, if the tumefaction or other conditions should prevent more effectual means from being used. A straight rod placed over that portion of the axis which can be felt, would assist the determination of its extension, and this would lead either to the axilla, the acromial portion of the clavicle, or the dorsum of the scapula, according to the variety of the dislocation.

The next step should be to endeavor to *feel* the head itself, as distinctly as may be, for there can be no harm in multiplying our resources, and it is important that the new position of the bone be accurately determined. As already intimated, the head of the bone can be more distinctly felt in the axilla when the arm is elevated than when otherwise. If it be below the clavicle, it will be made more prominent by carrying the elbow forcibly backward; and, finally, if dislocated upon the scapula the elbow should, on the contrary, be forcibly carried forward, in order to increase the prominence of the head of the humerus.

I have thus endeavored to direct attention to what I deem an exceedingly important addition to our means of diagnosticating dislocations of the shoulder, and have done so as briefly as the nature of the subject would permit. I may have erred in claiming for my views any originality, for the "wise man" has long since said that "there is nothing new under the sun," and daily experience confirms the correctness of the apothegm. I have neither the leisure nor the inclination to thumb the numerous tomes of surgical literature for the purpose of settling this point. It is certain, however, that if the method of examination here recommended has ever been suggested before, it has not been insisted on by the systematic writers usually consulted by our practitioners. If I may, therefore, have simply revived and magnified the value of what was known before, so that the profession and humanity may derive any advantage from this paper, my object will have been attained.—*Southern Medical and Surgical Journal*.

